# Assessing the impact of human genome annotation choice on RNA-seq expression estimates

**DOI:** 10.1186/1471-2105-14-S11-S8

**Published:** 2013-09-13

**Authors:** Po-Yen Wu, John H Phan, May D Wang

**Affiliations:** 1 School of Electrical and Computer Engineering, Georgia Institute of Technology, Atlanta, GA 30332, USA; 2 The Wallace H Coulter Department of Biomedical Engineering, Georgia Institute of Technology and Emory University, Atlanta, GA 30332, USA

## Abstract

**Background:**

Genome annotation is a crucial component of RNA-seq data analysis. Much effort has been devoted to producing an accurate and rational annotation of the human genome. An annotated genome provides a comprehensive catalogue of genomic functional elements. Currently, at least six human genome annotations are publicly available, including AceView Genes, Ensembl Genes, H-InvDB Genes, RefSeq Genes, UCSC Known Genes, and Vega Genes. Characteristics of these annotations differ because of variations in annotation strategies and information sources. When performing RNA-seq data analysis, researchers need to choose a genome annotation. However, the effect of genome annotation choice on downstream RNA-seq expression estimates is still unclear. This study (1) investigates the effect of different genome annotations on RNA-seq quantification and (2) provides guidelines for choosing a genome annotation based on research focus.

**Results:**

We define the complexity of human genome annotations in terms of the number of genes, isoforms, and exons. This definition facilitates an investigation of potential relationships between complexity and variations in RNA-seq quantification. We apply several evaluation metrics to demonstrate the impact of genome annotation choice on RNA-seq expression estimates. In the mapping stage, the least complex genome annotation, RefSeq Genes, appears to have the highest percentage of uniquely mapped short sequence reads. In the quantification stage, RefSeq Genes results in the most stable expression estimates in terms of the average coefficient of variation over all genes. Stable expression estimates in the quantification stage translate to accurate statistics for detecting differentially expressed genes. We observe that RefSeq Genes produces the most accurate fold-change measures with respect to a ground truth of RT-qPCR gene expression estimates.

**Conclusions:**

Based on the observed variations in the mapping, quantification, and differential expression calling stages, we demonstrate that the selection of human genome annotation results in different gene expression estimates. When conducting research that emphasizes reproducible and robust gene expression estimates, a less complex genome annotation may be preferred. However, simpler genome annotations may limit opportunities for identifying or characterizing novel transcriptional or regulatory mechanisms. When conducting research that aims to be more exploratory, a more complex genome annotation may be preferred.

## Background

Next-generation sequencing (NGS) technology is a powerful tool for extracting and interpreting genetic information from a broad range of biological systems, e.g., miRNA regulatory networks [1], genome-wide association between single nucleotide polymorphisms (SNPs) and phenotypes [2], DNA-protein interactions [3,4], and differentially expressed genes between treated and control samples [5,6]. NGS is preferable over first-generation Sanger sequencing because of its high sequencing throughput and low cost per base pair. With NGS, sequencing an entire human genome becomes feasible, which enables a larger cohort of samples in genome-scale comparative studies. RNA-seq is a major branch of NGS technology that is useful for studying transcriptomes [7]. One aspect of transcriptome research is quantification of expression levels for various genomic elements, e.g., genes, transcripts, and non-coding RNAs [8]. Acquiring transcriptome expression profiles requires that genomic elements be defined in the context of the genome. Multiple human genome annotations exist, including the AceView database [9] and the RefSeq database [10]. Thus, it is necessary to study the impact of genome annotation choice on transcriptome quantification.

Genome annotation is a dynamic process that defines coordinates for each genomic element with respect to the genome sequence. Such a process bridges the gap between DNA or RNA sequences and biological functions [11]. Integration of a genome annotation with mapping information from RNA-seq short sequence reads enables quantification of genomic elements such as genes and transcripts. Each genome annotation project uses different annotation strategies and information sources. Thus, high variation exists among multiple available annotations in terms of the comprehensiveness of annotated genomic elements. Some annotation strategies rely on computer-based prediction, resulting in more complex gene models that contain more predictive or exploratory genomic elements. Other annotation strategies rely on evidence-based methods, i.e., methods that require more manual curation, leading to simpler gene models with fewer genes and isoforms (i.e., splice variants of a gene).

We compare six human genome annotations from various databases, including the AceView database [9], the Ensembl database [12], the H-InvDB database [13], the RefSeq database [10], the UCSC Known Genes database [14], and the Vega database [15]. The key characteristics of each genome annotation are summarized in Table 1, in which annotations are ordered by decreasing complexity from left to right. The term "complexity" describes the primary differentiating characteristic among the genome annotations. We define the complexity of a human genome annotation to be proportional to the number of genes, isoforms, and exons. This definition enables us to investigate the relationship between downstream RNA-seq analyses (i.e., short sequence read mapping, gene expression quantification, and detection of differentially expressed genes) and the observed genome annotation complexity. We hypothesize that a more complex genome annotation is more difficult for RNA-seq mapping and quantification because of the difficulties of determining a best possible mapping from multiple candidate mappings and assigning unresolved ambiguous mappings to their correct genomic elements.

**Table 1:** Properties of various human genome annotations.

	Genome Annotations					
	
	*AceView Genes*	*H-InvDB Genes*	*Ensembl Genes*	*VegaGenes*	*UCSCKnown Genes*	*RefSeqGenes*
Version	2010	8.0	67	48	-	-
Database Downloaded Date	Nov. 12, 2011	Apr. 20, 2012	May 1, 2012	June 26, 2012	Dec. 21, 2011	July 23, 2012
# of Genes	72,376	43,893	48,817	44,880	28,423	23,731
# of Isoforms	259,426	236,861	177,858	158,835	75,725	41,099
# of Exons	678,503	542,099	534,400	493,509	273,711	227,710
Average # of Isoforms per Gene	3.58	5.40	3.64	3.54	2.66	1.73
Maximum # of Isoforms per Gene (with HGNC Gene Name)	119 (TRAV37)	885 (EEF1A1)	82 (GPR56)	77 (NDRG2)	129 (UTY)	77 (UTY)
Annotated Percentage (%) Gene	52.93	45.09	49.61	48.29	44.28	40.17
Exon	5.70	3.72	3.63	3.53	2.70	2.27
Coding Sequence	1.71	1.43	1.14	1.05	1.13	1.07

The annotated percentage is the total length of all genomic elements (gene, exon, or coding sequence) over the entire length of the human genome

Any relationship between genome annotation complexity and gene expression quantification could be helpful in guiding the selection of a genome annotation for various expression studies using RNA-seq data. Currently no guidelines for selecting a genome annotation for RNA-seq are available, and the effect of genome annotation choice on downstream data analysis is still unclear. The focus of this study is to acquire some insights into the impact of human genome annotation choice on RNA-seq expression estimates.

## Results and discussion

### Complexity of human genome annotations

**Figure 1:**
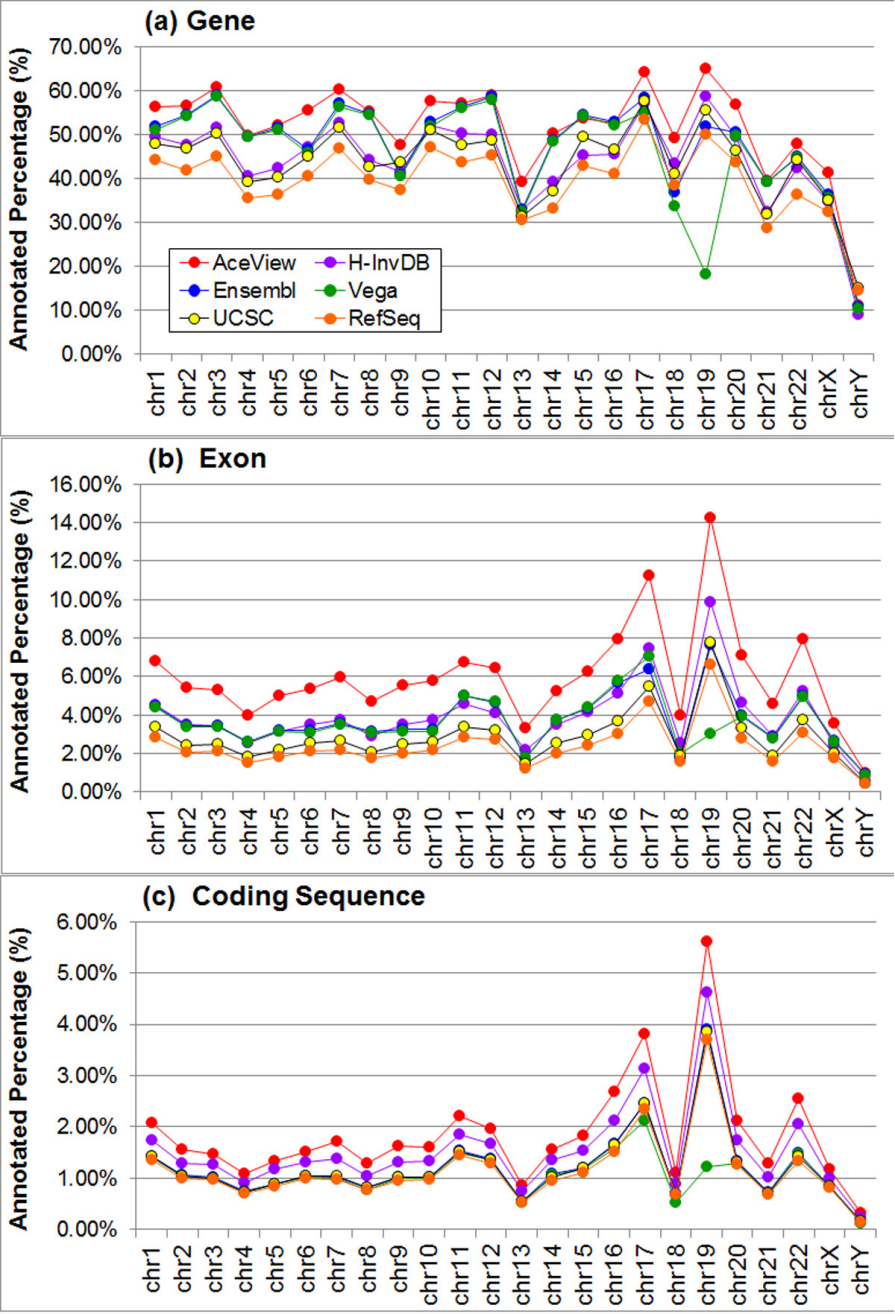
**Annotated percentage per chromosome of each genome annotation**. For each genome annotation, the annotated percentage of each chromosome is demonstrated on (a) the gene level, (b) the exon level, and (c) the coding sequence level. The AceView annotation usually has the highest annotated percentage for all chromosomes and all levels of comparison.

Table 1 summarizes several important statistics for each genome annotation. We rank the genome annotations based on the number of genes, isoforms, and exons. Ranking the set of human genome annotations (i.e., AceView, H-InvDB, Ensembl, Vega, UCSC, and RefSeq) by decreasing number of genes results in ranks of (1, 4, 2, 3, 5, 6). In other words, AceView is ranked at 1 because it has the largest number of genes while H-InvDB is ranked at 4. Similarly, ranking the set of human genome annotations by decreasing number of isoforms and exons results in identical ranks of (1, 2, 3, 4, 5, 6) and (1, 2, 3, 4, 5, 6), respectively. We then define the complexity rank of the genome annotation to be proportional to the average of these three ranks. The average ranks of these genome annotations are (1, 2.67, 2.67, 3.67, 5, 6). We use the mode of ranks to break ties (e.g., the H-InvDB and Ensembl annotations both have average ranks of 2.67). Thus, the human genome annotations are ordered by decreasing complexity as AceView, H-InvDB, Ensembl, Vega, UCSC, and RefSeq. The annotated percentage of each genome annotation generally follows the trend of complexity as demonstrated in Figure 1. For the average number of isoforms per gene and the maximum number of isoforms per gene, annotations generally have the same trend as the complexity measure. However, the H-InvDB annotation deviates from this trend, containing on average 50% more isoforms per gene compared to the most complex AceView annotation.

### Effect of human genome annotation complexity on mapping

**Figure 2:**
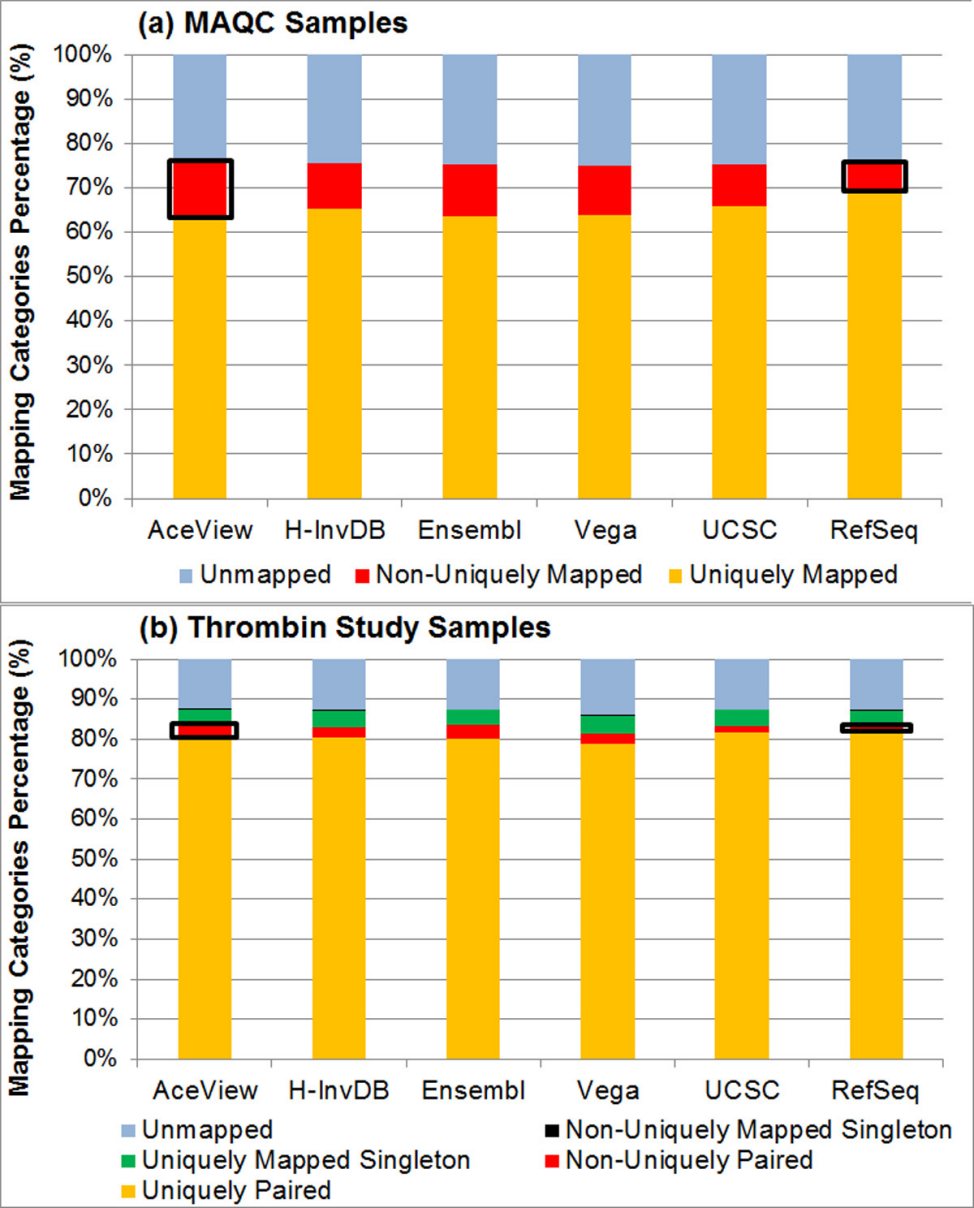
**Distribution of read mapping categories**. (a) MAQC samples (SRA: SRP000727) contain single-end reads, thus, there are three read mapping categories: uniquely mapped reads, non-uniquely mapped reads, and unmapped reads (b) Thrombin study samples (SRA: SRP008482) contain paired-end reads, thus, five read mapping categories can possibly occur. Cases of uniquely paired reads and non-uniquely paired reads occur when both ends of a read pair are mappable to the genome. Situations of uniquely mapped singletons and non-uniquely mapped singletons occur when only one end of a read pair is mappable to the genome. The RefSeq annotation has the highest percentage of uniquely mapped reads and the lowest non-uniquely mapped reads for both samples.

We propose two metrics to assess the effect of genome annotation complexity on sequence mapping. We first examine read mapping information and classify them into three categories for single-end sequencing samples or into five categories for paired-end sequencing samples. We use OSA alignment outputs as an example to demonstrate the impact of genome annotation choice on read mapping. For both the shorter read length single-end sequencing sample (1 × 36 bp; SRA: SRP000727) and the longer read length paired-end sequencing sample (2 × 100 bp; SRA: SRP008482), we observe similar results (Figure 2). The RefSeq annotation consistently has the highest percentage of uniquely mapped reads and uniquely paired reads in the single-end case and paired-end case, respectively. Note that the percentage of unmapped reads is similar for all annotations. The percentage of non-uniquely mapped reads or read pairs increases as genome annotation becomes more complex. Outlying cases exist (e.g., the Vega annotation has the lowest percentage of uniquely paired reads in paired-end sequencing samples), but the observed trend still follows the complexity measure. From Table 1, more complex annotations generally annotate more genes and isoforms and thus, increase the possibility of ambiguous mappings. These ambiguous mappings are more difficult to resolve for identifying the best mapping, which directly translates to the increase in percentage of non-uniquely mapped reads when using more complex annotations.

**Figure 3:**
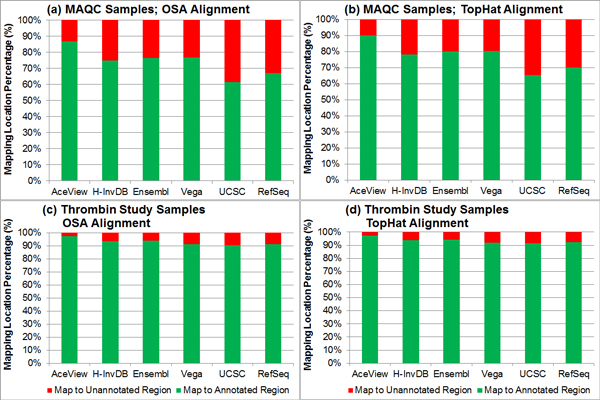
**The percentage of reads or read pairs that map to the annotated and un-annotated genomic sequences**. Sub-figures (a)-(d) represent different combinations of samples (top: MAQC samples with SRA accession number SRP000727; bottom: thrombin study samples with SRA accession number SRP008482) and spliced mappers (left: OSA; right: TopHat). The UCSC annotation usually has the lowest percentage of reads that mapped to the annotated genomic sequences, while the AceView annotation usually has the highest percentage. The same observation is applicable to all four combinations of samples and spliced mappers.

We then examine the percentage of reads that map to the annotated and un-annotated genomic sequences. More reads mapping to the annotated genomic sequences implies that more sequence information will be available for the quantification step. Reads mapping to the un-annotated regions are not useful for quantifying predefined genomic elements. From Figure 3, we observe that the AceView annotation results in the highest percentage of reads that map to annotated sequences. In contrast, the UCSC and RefSeq annotations have lower percentages of reads that map to annotated sequences, with UCSC being the lowest. Other than this outlying case, this evaluation metric follows the annotation complexity measure.

### Effect of human genome annotation complexity on quantification

**Figure 4:**
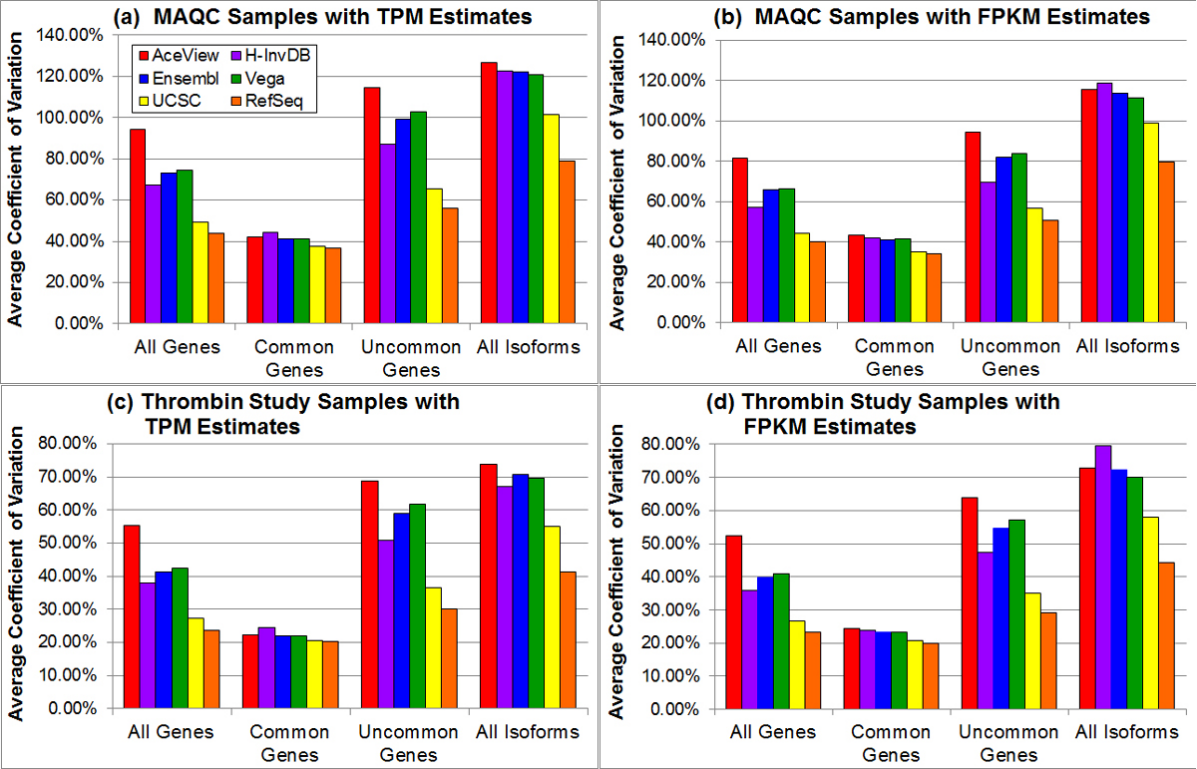
**The average coefficient of variation varies with different annotations and gene or isoform subgroups**. Sub-figures (a)-(d) represent different combinations of samples (top: MAQC samples with SRA accession number SRP000727; bottom: thrombin study samples with SRA accession number SRP008482) and expression estimates (left: TPM estimates from OSA package; right: FPKM estimates from TopHat alignment with Cufflinks quantification). The RefSeq annotation always has the smallest average coefficient of variation, while the AceView annotation has the highest average coefficient of variation for most of the cases. The variation is small when focusing on only common genes.

We propose two metrics to assess the impact of genome annotation complexity on RNA-seq quantification. The first metric is to evaluate the stability of gene and isoform expression estimates. Figure 4 demonstrates the variation of average coefficient of variation (CV) due to the choice of annotation and the selection of gene or isoform subgroups. We focus on four subgroups: all genes of each annotation, common genes (13,613 genes from the OSA pipeline and 13,810 genes from the TopHat/Cufflinks pipeline) that are defined in all annotations, genes not common to all annotations (i.e., uncommon genes), and all isoforms. The smaller variance between replicate expression estimates leads to the lower average CV. Trends for all genes and uncommon genes are similar. The AceView annotation has the highest average CV, followed by the Vega annotation, the Ensembl annotation, the H-InvDB annotation, the UCSC annotation, and the RefSeq annotation. In the case of all isoforms, sometimes the H-InvDB annotation results in the highest average CV. For common genes, the difference in average CV between various annotations is not large. The RefSeq annotation always results in the lowest average CV, whereas the H-InvDB or AceView annotations have the highest average CV. The variation between annotations becomes larger for the cases of all genes, uncommon genes, and all isoforms since more annotation-specific elements are being considered. More complex annotations are more challenging for quantification because a larger number of ambiguous mappings occur. Note that Ensembl and Vega deviate from the trend of the annotation complexity measure. A possible rationale for this observation is that the Ensembl and Vega annotations tend to include more small RNAs compared to the other annotations. Since the sequencing data we are analyzing follows the poly-A enrichment library preparation protocol, ideally, only mRNA is retained in the final sequencing samples. Thus, the majority of small RNAs should have zero or very low expression. We define these zero expressing elements as absent genomic elements. The inclusion of additional low expressing genomic elements in the Ensembl or Vega annotation results in larger average CV.

**Figure 5:**
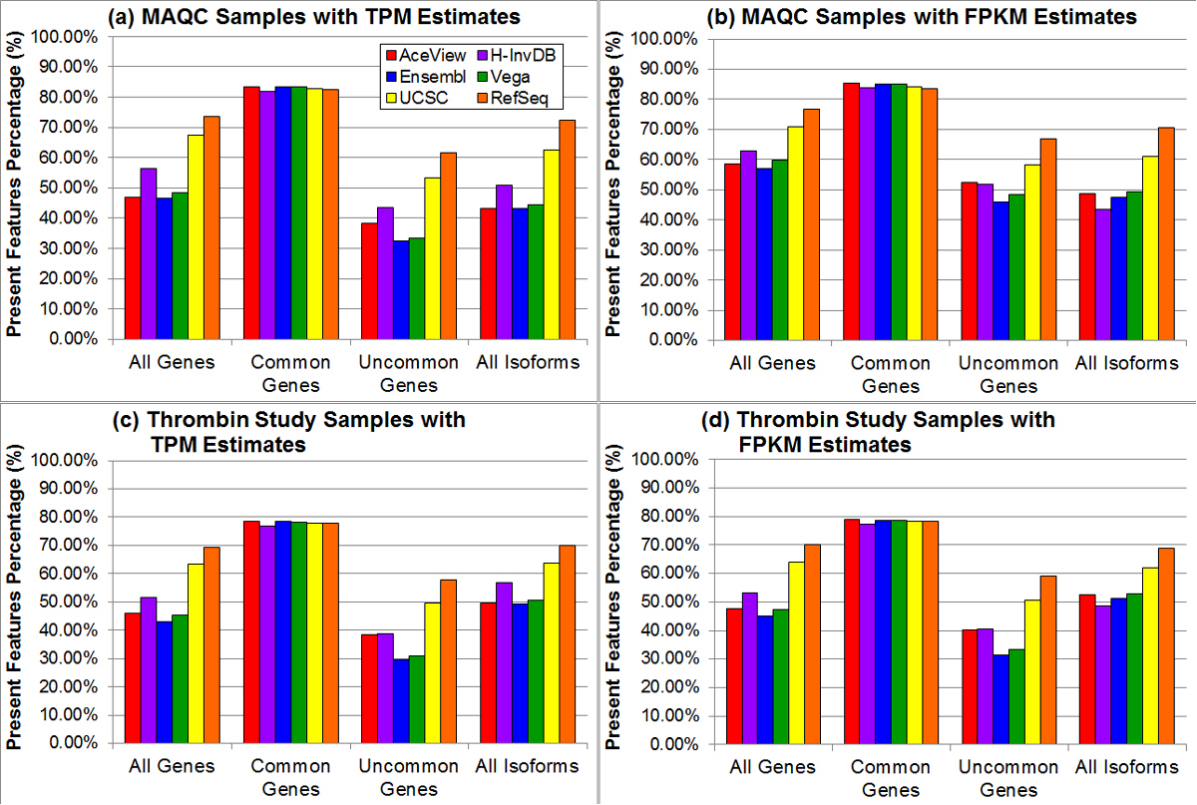
**The percentage of present genomic elements varies with different annotations and gene or isoform subgroups**. Sub-figures (a)-(d) represent different combinations of samples (top: MAQC samples with SRA accession number SRP000727; bottom: thrombin study samples with SRA accession number SRP008482) and expression estimates (left: TPM estimates from OSA package; right: FPKM estimates from TopHat alignment with Cufflinks quantification). The RefSeq annotation usually has the highest percentage of present genomic elements, while the Ensembl or Vega annotation generally has the lowest percentage of present genomic elements. The variation is small when focusing on only common genes.

Figure 5 demonstrates that the percentage of present genomic elements depends on the annotation. We define a 'present' genomic element to be an element that has nonzero expression for at least one technical replicate. For common genes, all annotations have a similar percentage of present genes. For uncommon genes, all genes, and all isoforms, the relation among the AceView, H-InvDB, Ensembl, and Vega annotations is more uncertain compared to other evaluation metrics. In most cases, the H-InvDB annotation has a higher percentage of present genes/isoforms than the AceView annotation. The RefSeq annotation always has the highest percentage of present genes/isoforms, followed by the UCSC annotation. As we explained earlier, more small RNAs are included in the Ensembl and Vega annotations. Because of the poly-A enrichment library preparation, most of these small RNAs have zero expression and are identified as absent, which correspondingly decreases the percentage of present genes or present isoforms.

### Effect of annotation complexity on differential expression calling

For thrombin study samples (SRA: SRP008482), we use OSA alignment with htseq-count quantification to prepare input read count data for the edgeR package. Most of the top 20 differentially expressed genes are detected in at least two of the six annotations, while several others are unique and annotation-specific [16]. Even though the functions of these annotation-specific genes are unclear, more complex annotations are still preferable since they provide an opportunity to identify novel genomic elements that may be functionally important.

Three genes were validated by RT-qPCR technology for the thrombin study samples (SRA: SRP008482). We examine the difference between RNA-seq-based fold-changes and RT-qPCR-based fold-changes and summarize the results in Table 2. From Table 2, we observe that the UCSC annotation always outperforms RefSeq annotation in terms of the lowest average absolute deviation from the RT-qPCR fold-change estimates. However, the difference between them is not large. In contrast, AceView and H-InvDB annotations have relatively higher average absolute deviations. We can infer from this observation that more complex annotations increase the difficulty of estimating gene expression accurately. Higher variations in gene expression estimates propagate to fold-change estimates and other differential expression test-statistics.

**Table 2:** Fold-changes between thrombin-treated samples and control samples (SRA: SRP008482).

		RNA-seq data with TPM Expression Estimates (OSA Pipeline)					
**Gene**	**RT-qPCR**	**AceView**	**H-InvDB**	**Ensembl**	**Vega**	**UCSC**	**RefSeq**

TRAF1	2.862	3.029	3.034	3.025	2.998	2.934	2.922
FANCD2	-1.050	-0.782	-0.687	-0.888	-0.856	-0.840	-0.840
CELF1	-0.202	-0.138	-0.202	-0.098	-0.098	-0.239	-0.275
Average Absolute Deviation from RT-qPCR		0.166	0.178	0.143	0.145	0.106	0.114

		**RNA-seq data with FPKM Expression Estimates (TopHat/Cufflinks Pipeline)**					

**Gene**	**RT-qPCR**	**AceView**	**H-InvDB**	**Ensembl**	**Vega**	**UCSC**	**RefSeq**

TRAF1	2.862	3.874	3.845	3.797	3.719	3.677	3.674
FANCD2	-1.050	0.057	0.057	-0.345	-0.322	-0.202	-0.151
CELF1	-0.202	0.642	0.516	0.595	0.585	0.390	0.356
Average Absolute Deviation from RT-qPCR		0.987	0.936	0.812	0.791	0.751	0.756

**Figure 6:**
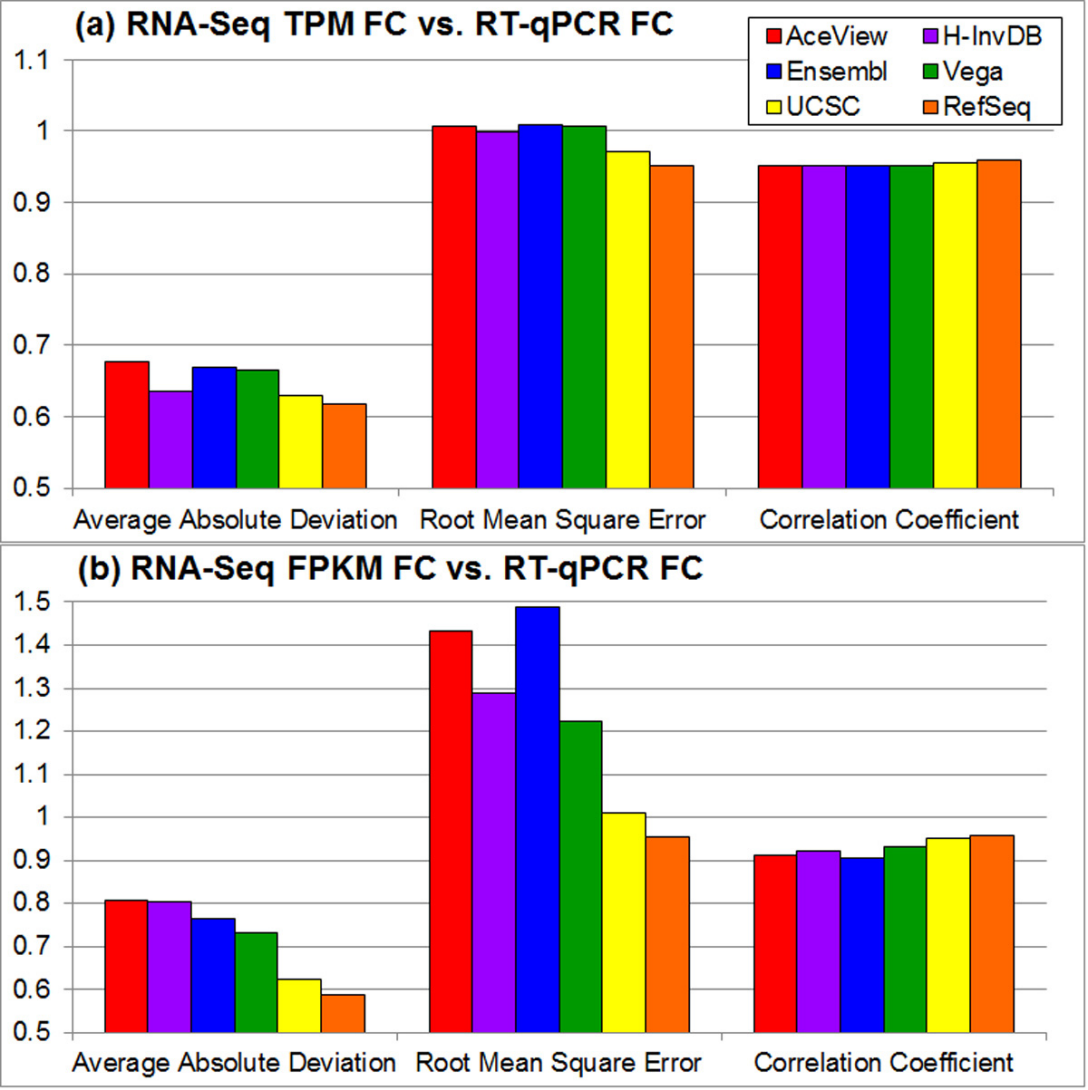
**Statistics for comparing RNA-seq-based fold-changes and RT-qPCR-based fold-changes vary with different annotations**. The comparison of fold-change estimates between RNA-seq and RT-qPCR using two RNA-seq expression estimates and three statistics. (a) TPM estimates are produced by the OSA package. (b) FPKM estimates are generated by Cufflinks with TopHat alignment. The RefSeq annotation always has the lowest (1) average absolute deviation and (2) root mean square error, and the highest correlation coefficient when treating RT-qPCR estimates as the ground truth.

The RT-qPCR data for MAQC samples is publicly available in the GEO database. We use three statistics to assess variations due to genome annotation choice. As shown in Figure 6, less complex genome annotations, e.g., the RefSeq annotation, results in lower average absolute deviation, lower root mean squared error (RMSE), and higher correlation coefficient when comparing RNA-seq fold-change estimates to RT-qPCR fold-change estimates. The variation between annotations is larger when using FPKM expression estimates from the TopHat/Cufflinks pipeline. Some outlying cases exist (e.g., the Ensembl annotation has the highest RMSE when using FPKM expression estimates), but the general trend of this evaluation metric still follows the annotation complexity measure.

## Conclusions

The genome annotation is a necessary component of RNA-seq expression analysis. Multiple genome annotations are publicly available; however, it is not clear how different choices of genome annotation will affect downstream RNA-seq expression estimates. We defined the complexity of human genome annotation, and assessed the relationship between genome annotation complexity and several RNA-seq performance metrics. Based on our complexity measure, we ordered existing human genome annotations from most to least complex as follows: AceView, H-InvDB, Ensembl, Vega, UCSC, and RefSeq. In more complex annotations, a higher percentage of the entire genome is annotated. For RNA-seq sequence mapping, less complex annotations result in a higher percentage of uniquely mapped reads and uniquely mapped pairs for both single-end and paired-end sequencing samples. However, at the same time, the number of RNA-seq reads that map to annotated genomic sequences is smaller for less complex annotations. Genome annotation complexity also affects RNA-seq expression estimates. More complex annotations result in more ambiguous mappings, which increase the difficulty of RNA-seq quantification and thus, cause higher expression variation between RNA-seq technical replicates. Furthermore, more complex annotations lead to a lower percentage of present (i.e., detected) genes or isoforms, which suggests that the predictive or hypothetical genomic elements in these annotations tend to belong to non-expressors or low expressors. For RNA-seq differentially expressed gene detection, the concordance is high among the six annotations. More complex annotations are capable of identifying annotation-specific genes that may be functionally important. Deviations in RNA-seq expression estimates due to differences in genome annotation complexity can propagate to fold-change statistics and, subsequently, differential expression detection. Thus, when comparing RNA-seq fold-change statistics to ground truth RT-qPCR fold-change statistics, more complex annotations tend to have larger deviation and smaller correlation. In summary, the impact of genome annotation choice on RNA-seq expression estimates is significant, and the choice of annotation should depend on the study. Less complex genome annotations are preferable for studies that require more stable RNA-seq expression estimates. However, to discover and explain unknown biological mechanisms, more comprehensive and complex genome annotations are necessary.

## Methods

### Study overview

**Figure 7:**
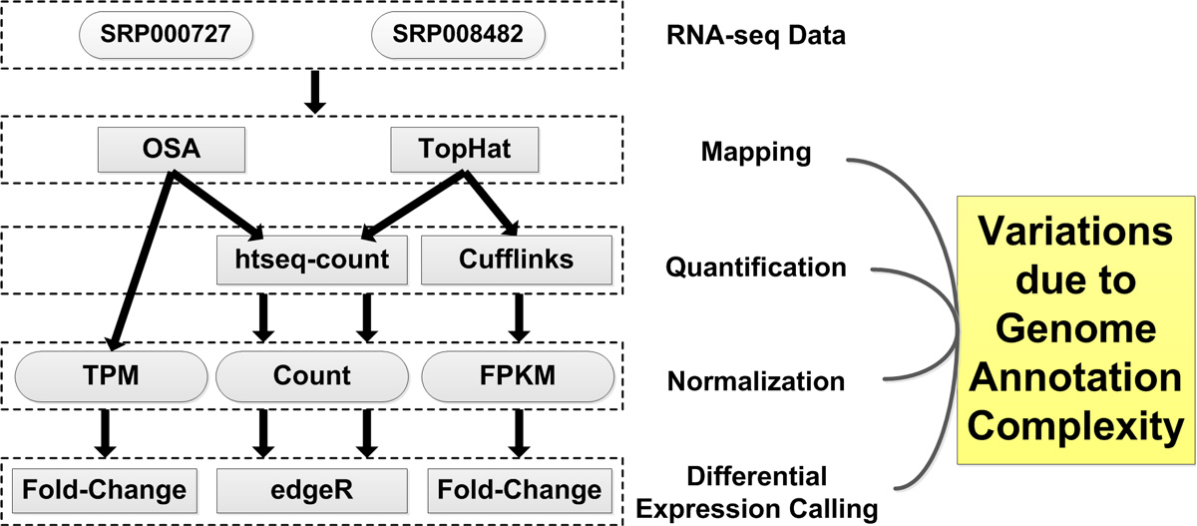
**Workflow of RNA-seq data analysis pipeline**. The five dashed boxes correspond to five steps in the RNA-seq data analysis pipeline. We applied two alignment tools and two quantification tools to estimate gene/isoform expression with normalization methods of count, TPM, or FPKM. The fold-change method and the edgeR tool are used to infer differentially expressed genes. At each analysis step, we assess the variations resulting from genome annotation choice.

This paper aims to provide insights into the effect of different choices of human genome annotation on the variation in RNA-seq expression estimates. We propose a complexity measure (refer to the Background section) to relate observations in downstream RNA-seq analyses to the trend of genome annotation characteristics. The typical pipeline for RNA-seq expression analysis includes mapping, quantification, normalization, and calling differentially expressed genes. As shown in Figure 7, in this study, we use two publicly available RNA-seq datasets that provide differentially expressed genes and RT-qPCR validation information. We align short sequence reads to the human genome with two spliced aligners, OSA [17] and TopHat [18]. Alignment outputs of both tools are quantified by htseq-count [19] to acquire gene expression estimates in terms of the read counts. Since OSA has embedded quantification and TPM normalization [20] in its package, we use Cufflinks [21] to quantify TopHat alignment outputs only and then obtain gene/isoform expressions in terms of FPKM-normalized values [8]. Given the read counts data from htseq-count, we apply the edgeR package in R [22] to call differentially expressed genes between treatment and control samples. For TPM or FPKM expression estimates, we calculate fold-change between treatment and control samples and then compare these numbers to external RT-qPCR validation results provided by the original studies. We propose several evaluation metrics for each analysis step to demonstrate performance variation induced by annotation complexity.

### Human genome annotation

Several organizations or institutions have spent more than a decade working on annotating the human genome. Various annotating techniques have been developed and a variety of information sources have been utilized to provide the most informative and correct human genome annotation [23,24]. We use six well-known annotations, including AceView Genes, Ensembl Genes, H-InvDB Genes, RefSeq Genes, UCSC Known Genes, and Vega Genes. Since each annotation emphasizes slightly different categories of genomic elements (e.g., some report more functionally important small RNAs), we need to manually examine these annotations and prepare them to be comparable with each other. We choose to use AceView annotation as a standard to determine which genomic elements need to be filtered out in other annotations. We also decided to use annotations that only fall into main chromosome contigs, unplaced contigs, and unlocalized contigs in the UCSC HG19 assembly.

The information sources and annotating strategies of each human genome annotation are summarized in [16]. The procedures for improving cross-annotation comparability are briefly described below:

AceView Genes [9] - The AceView annotation was downloaded from its website. We choose this annotation as the standard definition for genomic element categories. To match genomic contigs for the sequence mapping stage, we remove the mitochondrial annotation from the original annotation file.

Ensembl Genes [12] - The Ensembl annotation was downloaded from its FTP site. It includes annotations that fall outside of the preselected genomic contigs as well as a considerable amount of small RNAs (e.g., tRNA, miscRNA scRNA, snRNA). These genomic elements are removed in the preparation process. Meanwhile, the chromosome names in this annotation are translated to the chromosome names in HG19 convention.

H-InvDB Genes [13] - The H-InvDB annotation was downloaded from its website. To prepare this annotation, we remove annotations located on haplotype and mitochondrial contigs. We also map the chromosome names in H-InvDB annotation to the chromosome names in HG19 convention.

RefSeq Genes [10] - The RefSeq annotation was downloaded from the UCSC Table Browser. We prepare the RefSeq annotation by removing haplotype annotations.

UCSC Known Genes [14] - The UCSC Known Genes annotation was downloaded from the UCSC Table Browser. To prepare this annotation, we remove annotations located on the haplotype and mitochondrial contigs. We also remove other small RNAs such as tRNA, miscRNA, and snRNA.

Vega Genes [15] - The Vega annotation was downloaded from its website. The preparation steps are similar to that of the Ensembl annotation. Genomic elements that fall outside of the preselected genomic contigs are removed. The chromosome names in Vega annotation are also translated to the chromosome names in HG19 convention.

### RNA-seq datasets

We download two publicly available RNA-seq datasets from the NCBI Sequence Read Archive (SRA) repository. The first dataset (accession number: SRP008482) investigates how thrombin treatment affects endothelial function in terms of gene expression profiles. Generally, thrombin can stimulate endothelial cells and regulate the expression, release and activation of a number of biological mediators [25]. The targeted biological samples are "human pulmonary microvascular endothelial cells (HMVEC-L)" with two conditions: either control (two technical replicates) or treated with thrombin for six hours (three technical replicates). The sequencing platform was Illumina HiScanSQ with sequencing depth around 50 million read pairs for each technical replicate, and read lengths of 2 × 101 base pairs. The study also validated the expression fold-change of three genes (CELF1, FANCD2, and TRAF1) between treated and control samples using RT-qPCR technology. Such RT-qPCR information is considered the ground truth and is useful for validating and evaluating RNA-seq expression estimates.

The second dataset (accession number: SRP000727) studies alternative isoform regulation in human tissue transcriptomes [26]. It profiled 16 tissue transcriptomes, and two MAQC (MicroArray Quality Control) samples were included in the study. The two MAQC samples are Ambion Human Brain Reference RNA (HBRR) and Stratagene Universal Human Reference RNA (UHRR). The study includes four technical replicates for the HBRR sample and 3 technical replicates for the UHRR sample. This is an older sequencing dataset which used Illumina Genome Analyzer to generate single-end reads with read length of 36 base pairs. Each technical replicate has only 2.5 million reads. The merit of this dataset is that the RT-qPCR results are publicly available through the MAQC study. Fold-changes of 1,044 genes from the TaqMan RT-qPCR assay again provide an external ground truth for evaluating RNA-seq expression estimates.

### Short sequence read mapping methods and evaluation metrics

**Figure 8:**
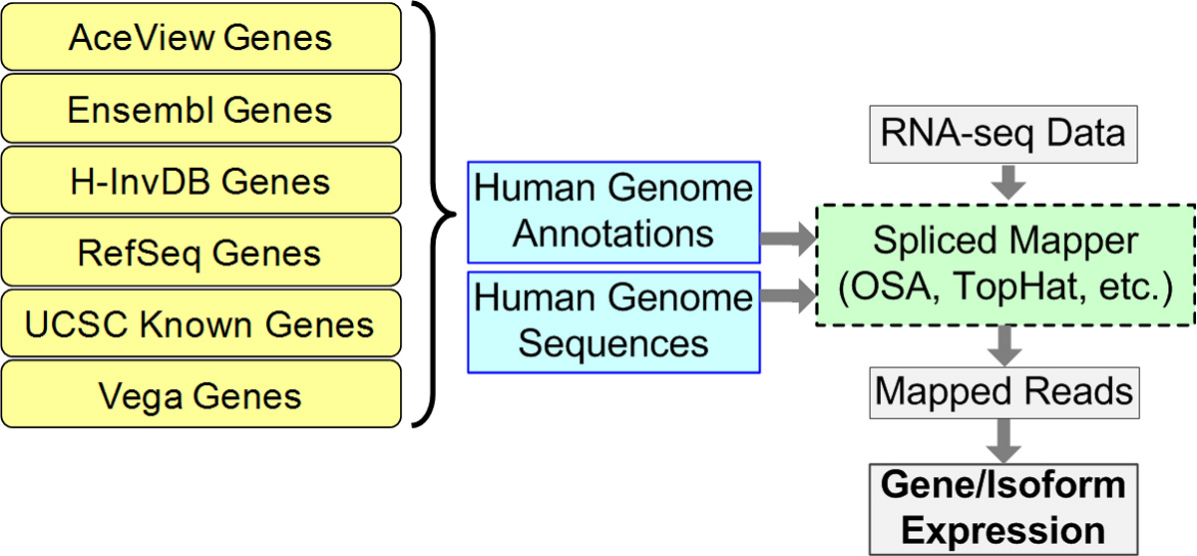
**Workflow of typical RNA-seq spliced alignment pipeline**. A spliced mapper aligns RNA-seq data to a genome with the help of a genome annotation. Different genome annotations define various sets of exon junction information which affect the output of spliced mappers. The mapped reads can then be used to quantify gene/isoform expressions.

We use two spliced alignment tools, TopHat and OSA, to map short sequencing reads to the human genome with the guidance of various genome annotations. The spliced alignment tools enable RNA-seq reads to directly map to the genome (the typical pipeline for spliced alignment is shown in Figure 8). TopHat is a spliced alignment tool that is widely used for mapping RNA-seq data to the genome or transcriptome [18]. It aligns short sequence reads to the human transcriptome first, and then attempts to remap the unmapped reads from the previous stage to the human genome. The alignment outputs from the two stages are merged into the final output. We use TopHat version 2.0.5 with no novel junction detection and the "-G" option, which allows us to provide a genome annotation GTF file (the standard genome annotation reporting format) and to force TopHat to use the mapping strategy described in Figure 8. OSA (Omicsoft Sequence Aligner) is a new spliced alignment tool that "improves mapping speed 4-10-fold with better sensitivity and less false positives" compared to the TopHat, SoapSplice, and RUM pipelines [17]. It implements a similar mapping strategy as TopHat. We use OSA version 1.8.2 with the default settings.

We use the UCSC HG19 human genome assembly as a reference genome for spliced alignment. We include only 24 main chromosome contigs, 20 unplaced contigs, and 39 unlocalized contigs. The rest of the contigs, e.g., mitochondria and haplotypes, are excluded.

We define two evaluation metrics for the mapping stage. The first metric is based on the categorization of read mapping outcomes. For paired-end sequencing, the categories include uniquely paired reads, uniquely mapped singletons, non-uniquely paired reads, non-uniquely mapped singletons, and unmapped reads. For single-end sequencing, the categories are simpler, including only uniquely mapped reads, non-uniquely mapped reads, and unmapped reads. The second metric is the percentage of the number of reads that map to the annotated and un-annotated genomic sequences.

### Gene/isoform expression quantification, normalization methods, and evaluation metrics

We use the htseq-count script from the HTSeq package to count the number of reads (or fragments in the paired-end sequencing case) that map to each gene as the gene expression estimate. For each mapped read or fragment, htseq-count determines the genes to which these reads or fragments associate. If a read or a fragment overlaps more than one gene, it provides three scenarios to resolve this ambiguous situation. We use the HTSeq package version 0.5.3p9 with "intersection-nonempty" overlapping resolution [19]. All the other options were kept in the default setting. Read counts from htseq-count are used as the input for packages that detect differentially expressed genes. The OSA package includes functionality for quantifying and normalizing gene/isoform expressions. We choose to report gene/isoform expression estimates in terms of Transcripts Per Million (TPM) normalized values [20]. For TopHat alignment outputs, we use Cufflinks to quantify gene and isoform expressions in terms of Fragments Per Kilobase exon model per Million mapped reads (FPKM) estimates. We use Cufflinks version 2.0.2, keeping most parameters in the default setting except enabling sequencing bias correction and multi-mapped reads correction [21].

At the quantification and normalization stage, we propose two evaluation metrics to observe the impact of genome annotation complexity on RNA-seq expression estimates. We use the stability of normalized TPM or FPKM expression between technical replicates as the first evaluation metric. With the help of unique HGNC gene identifier for each gene, we identify 13,613 common genes from OSA alignment with TPM quantification and 13,810 common genes from TopHat alignment with Cufflinks FPKM quantification across six genome annotations. The number of common genes between the two pipelines is different since Cufflinks tend to collapse or not report certain low-expressing genes. For each genome annotation, we remove genes that are absent for all replicates and then compute the coefficient of variation (CV), averaged across all targeted genes or isoforms, as defined in equation (1), where S_i _is the sample standard deviation of expression estimates across replicates with the same biological condition, ${\bar{x}}_{i}$ is the mean of expression estimates across replicates with the same biological condition, and *n *is the number of targeted genes or isoforms. We apply the same technique to the set of common genes, uncommon genes, and to all isoforms.

(1) $$Average\;CV=\left[\;\frac{1}{n}\;\left({\left(\overset{n}{\underset{i=1}{\sum }}S_{i}\;/\;{\bar{x}}_{i}\;\right|}_{condition\;\;1}\right)+\frac{1}{n}\;\left({\left(\overset{n}{\underset{i=1}{\sum }}S_{i}\;/\;)\;{\bar{x}}_{i}\;\right|}_{condition\;\;2}\right)\;\right]/)\;2$$

The second evaluation metric is the percentage of present genes or isoforms that have nonzero expression in at least one replicate. Since some annotations (e.g., the AceView annotation) possess considerably more genes or isoforms compared to other annotations, through this metric, we aim to examine the practicability of this additional information for transcriptome profiling studies (assuming sequencing libraries are prepared by poly-A enrichment).

### Differentially expressed gene calling methods and evaluation metrics

The edgeR package in R is applied to identify differentially expressed genes [22]. It takes the raw read count as the input instead of the normalized expression estimates. For each gene, edgeR assumes that the raw read count across several replicates follows a negative binomial distribution. The Fisher's exact test is chosen to determine the significance level of each differentially expressed gene (DEG). To examine the repeatability of DEGs, we observe the concordance of the 20 most significant DEGs from each annotation.

The TPM and FPKM expression estimates are used for fold-change evaluation. For thrombin study samples (SRA: SRP008482), three genes were selected for RT-qPCR validation with fold-changes provided in the original study. For each of the six annotations, we consider the RT-qPCR result as a ground truth for estimating the error of RNA-seq-based fold-changes from RT-qPCR-based fold-changes. We use the average absolute deviation to measure the error as defined in equation (2), where FC stands for Fold-Change and *n *is the number of fold-changes being compared. We use Log_2 _transformation for the fold-change estimates for both technologies.

(2) $$Average\;Absolute\;Deviation=\frac{\sum_{n}\left|FC_{RNA-seq}-FC_{RT-qPCR}\right|}{n}$$

For MAQC samples (SRA: SRP000727), we use the publicly available MAQC-I dataset that used a TaqMan RT-qPCR assay to profile 1,044 genes for both the Human Brain Reference RNA (HBRR) and Universal Human Reference RNA (UHRR) samples [27] (GEO accession number: GSE5350). There are four technical replicates for the HBRR sample and four technical replicates for the UHRR sample. We select 830 TaqMan genes that have present calls for all replicates of HBRR and UHRR samples, and then calculate (1) average absolute deviation, (2) root mean squared error, and (3) correlation coefficient between RNA-seq-based fold-changes and RT-qPCR-based fold-changes. We use Log_2 _transformation for the fold-change estimates for both technologies.

## Competing interests

The authors declare that they have no competing interests.

## Authors' contributions

PW designed and executed the computational experiments to compare the effect of different human genome annotations on RNA-seq analysis and drafted the manuscript. JHP contributed to the design of performance metrics, selection of datasets, and revision of the manuscript. MDW initiated the RNA-seq factorial analysis, acquired various funding to sponsor the project, and directed publication, including contributions to manuscript preparation and revision. All authors read and approved the final manuscript.
